# Effect of Femoral Stem Modular Neck’s Material on Metal Ion Release

**DOI:** 10.2174/1874325001711011337

**Published:** 2017-11-29

**Authors:** Janie Barry, Marc-Olivier Kiss, Vincent Massé, Martin Lavigne, Jihad Matta, Pascal-Andre Vendittoli

**Affiliations:** Hôpital Maisonneuve-Rosemont, Université de Montréal, 5415 Boulevard de l’Assomption, Montreal, Canada

**Keywords:** Ceramic-on-ceramic, Metal ions, Modular neck, Total hip arthroplasty, Corrosion, Taper junction

## Abstract

**Background:**

In recent decades, the popularity of modular necks in total hip arthroplasty (THA) has increased since modular necks offer the potential to restore the patient’s native anatomy, and thus improve stability. Unfortunately, modular necks are associated with higher complication rates, including implant fracture and modular junction corrosion with adverse local tissue reaction to metal debris.

**Objective:**

The objective of this study was to determine the impact of modular neck material on titanium (Ti), chrome (Cr), and cobalt (Co) metal ion levels in patients who underwent a THA with Ti femoral stem, Ti or CrCo modular neck, and ceramic-on-ceramic (CoC) bearing.

**Methods:**

Whole blood Ti, Cr, and Co levels were compared at a minimum 1-year follow-up in 36 patients who underwent unilateral, primary CoC large-diameter head THA with Profemur^®^ Preserve modular femoral stems (MicroPort, Arlington, TX, USA).

**Results:**

Higher Co concentrations were observed in the CrCo modular neck group (0.46 versus 0.26 µg/l in the Ti neck group, P=0.004) and higher Ti concentrations were observed in the Ti modular neck group (1.98 *vs* 1.59 µg/l in the CrCo neck group, P=0.007). However, these differences were not clinically meaningful since the absolute values remained within what is considered the safe range of Ti, Cr, and Co ions in whole blood. No patients were re-operated or revised.

**Conclusion:**

Modular neck materials had an impact on whole blood metal ion levels but the concentrations measured remained within the safe range at 1-year follow-up. There were no indirect signs of modular junction corrosion with either CrCo or Ti femoral necks.

## 1 INTRODUCTION

Total hip arthroplasty (THA) is one of the most successful surgical procedures. Over the last few decades, much research has been carried out with the goal of increasing implant durability, reducing complications, and improving the capacity of the implant to reproduce native hip anatomy and function. Modular necks, introduced in 1970, have surged in popularity in recent decades as they offer surgeons the potential to enhance hip stability by restoring the native anatomy of the patient’s hip [1]. However, some types of hip implants are associated with high complication rates, including modular junction corrosion, adverse local tissue reaction to metal debris (ARMD), and implant fracture [2-7]. In June 2012, Stryker issued a voluntary recall of its Rejuvenate and ABG II modular neck stems due to potential fretting and corrosion at the modular neck junction that resulted in ARMD, pain, and swelling around the hip joint [7-9].

The corrosion process that occurs at the neck-stem junction is not yet completely understood, although several factors have been identified. Galvanic corrosion, an electrochemical reaction, occurs when two dissimilar metals are in contact with one another. Fretting corrosion, on the other hand, is induced by highly-loaded metal surfaces that exist in a micromotion environment [8]. This kind of corrosion leads to crevice formation, which, in turn, allows aqueous material to accumulate and become stagnant. This ultimately gives rise to the migration of negatively charged particles that may elicit hydrochloric acid production, the latter being responsible for titanium (Ti), chromium (Cr) and cobalt (Co) alloy erosion [10]. Junction loading is a major factor related to corrosion. The use of large femoral heads (greater than 36 mm), with greater offset and longer neck length, may increase stress at the junction, leading to increased corrosion. It has been established that crevice corrosion is an irreversible process that persists, even after the load is reduced or eliminated. Corrosion is accompanied by the release of metal particles that induce ARMD, pseudo-tumor formation, and periprosthetic bone osteolysis [11, 12].

Although, it does not follow a linear relationship, measuring metal ion levels in blood is an indirect way to estimate the extent of ion release from metallic implants. Since corrosion liberates metal ions, its magnitude may be estimated by quantifying the metal ion concentration in the blood of patients [13, 14].

The objective of this study is to assess the impact of modular neck materials (Cr, Co and Ti) on metal ion levels in patients who underwent a THA with a modular neck made of Ti or CrCo and a CoC bearing.

## 2 MATERIALS AND METHODS

The study protocol was implemented after being approved by the local institutional ethics review board. It enrolled a consecutive cohort of 36 patients returning for follow-up assessment at least 1 year after a THA was performed using a large diameter head CoC bearing Maxera cup (Zimmer, Warsaw, IN, USA) coupled with the Profemur^®^ Preserve modular femoral stem (MicroPort, Arlington, TX, USA). Patients with other metallic implants (other joint replacement, open reduction internal fixation hardware, etc.) or renal insufficiency were excluded.

Profemur^®^ Preserve modular stems are short modular Ti femoral stems (ASTM F136 Titanium-6 Aluminum-4 Vanadium ELI alloy). Their proximal portion is plasma sprayed with Ti and their distal portion is grit-blasted. All patients received a Delta ceramic femoral head (distributed by MicroPort but manufactured by Ceram Tec GmbH, Plochingen, Germany). Implanted femoral heads had a diameter of 36 mm and 40 mm depending on the size of the acetabular component and did not have an adaptive Ti sleeve. Femoral head taper was 12/14, 5°46’ and, according to MicroPort, femoral stem trunnion taper conformed to Ceram Tec’s head taper. The first group of 22 patients received a Ti modular femoral neck (ASTM F136 Ti6Al4V ELI alloy) while the second group of 14 patients received a CoCr femoral neck (ASTM F1537 wrought CoCrMo alloy). Femoral stem pocket taper is not standard. Its size, angle, and surface finish were developed by Cremascoli in 1988, and is specific to MicroPort only: MPO proprietary taper. Using Ti femoral necks, the modular junction of this Ti femoral stem family (Profemur^®^) has been in use since 1985, and good clinical results have been reported [15]. In 2015, however, a failure rate of 6.6% was reported in a series of 277 subjects [16]. After these alarming results were published, the manufacturer decided to switch the material of the “long” neck to CrCo. The two groups in this study represent patients who underwent THA during the period of switching from Ti to CrCo for the “long” necks in our clinical practice. The difference in number between the Ti and CrCo neck groups (22 *vs* 14) corresponds to our utilization ratio of short/long neck.

All subjects received a Maxera monoblock acetabular component (Zimmer). This implant is made of Ti6Al4V alloy with Ti plasma sprayed on its outer surface. It has a Delta ceramic insert, assembled by the manufacturer (non-modular from a surgeon’s perspective). With this system, the bearing diameter is dictated by acetabular component size (46- and 48-mm components have 36-mm bearing diameter, and 50- and 52-mm components have 40-mm bearing diameter). Surgeries were performed *via* a posterior approach by two surgeons. Following the manufacturer’s recommendations, taper junctions (neck-stem and head-neck) were cleaned before impaction. The modular neck and femoral head were impacted separately, each with 3-4 hammer blows.

Whole-blood Co, Cr, and Ti ion levels were measured using standardized techniques in a single-blinded laboratory. A research nurse collected blood samples from all patients by cannulation with a 22-gauge stainless steel needle (BD Insyte, BD Medical, Salt Lake City, UT, USA), with the plastic cannula left in place and the metallic needle discarded. The first 5 ml of blood collected was discarded to avoid contamination from the metallic needle. Three 5-ml blood samples were then collected in BD Vacutainer Plus^®^ plastic whole blood tubes (Lavender BD Hemogard™ closure, reference 367861, K2EDTA spray-dried, 7.2 mg). Patients were excluded from further analysis if the sampling collection protocol was unsuccessful after two failed attempts. All samples were kept frozen at -20°C. They were analyzed by Trace Element Laboratories in London, Ontario, Canada. Ti ion concentrations were measured in a MAT Element 2 high-resolution, sector-field, inductively-coupled plasma mass spectrophotometer (Finnigan, Waltham, MA, USA). The detection accuracy is 0.1 μg/l Ti.

Early postoperative, 1-year, and last follow-up radiographs were analyzed for implant position and signs of loosening. Clinical function was evaluated with WOMAC (Western Ontario and McMaster Universities Osteoarthritis Index) [17] and UCLA (University of California at Los Angeles) activity scores [18].

Statistical analyses were performed using SPSS 21.0 (SPSS, Inc., Chicago, IL, USA). Continuous variables are presented as mean ± SD, and categorical variables, as frequency and percentages. Between-group differences in continuous variables, such as age, body mass index (BMI), follow-up time, WOMAC, and UCLA scores were assessed by independent t-test. Metal ion levels were analyzed by the non-parametric Mann-Whitney test. Fisher’s exact test compared gender and neck length (short versus long). Stem models were compared by Chi-square test. Relationships between metal ion levels and age, BMI, cup size and follow-up time were tested by Pearson correlations. Statistical significance was set at P<0.05.

## 3 RESULTS

Demographic data are presented in Table **1**. A significant age difference was observed between groups. For all patients, mean follow up for metal ion measurement, clinical score assessment, and radiographic review, was 20 months (min 9, max 44). Whole blood metal ion concentrations are reported in Table **2** and Fig. (**1**). Significant between-group differences were evident for Co and Ti levels only. No significant correlations were apparent between metal ion levels and follow-up time, BMI, cup size, age, gender, and neck length. The clinical scores were similar between groups, as shown in Table **3**. To date, no patient has undergone reoperation or revision. One patient in the Ti neck group reported occasional squeaking from his hip during intense activities or extreme range of motion. One patient in the CrCo neck group reported occasional clicking sounds.

## 4 DISCUSSION

Although, modular necks may offer advantages, including reproducing the patient’s native anatomy during THA, some concerns remain about mechanically-assisted crevice corrosion and galvanic corrosion of the additional modular junction, and/or neck fracture. The impact of modular neck materials on metal ion release was assessed as galvanic corrosion may increase when two different metals are in contact. The 2 groups of patients in our study underwent THA with the same femoral stem type and CoC bearing. The only difference in implants between the 2 groups was in the modular neck which was composed of CrCo in one group and Ti in the other. Thus, observed differences in whole blood metal ion concentrations was very likely related to the material found in the modular neck, allowing a better estimation of the performance of each metal at the femoral stem modular junction. Statistically significant differences in whole blood metal ion concentrations were noted between the 2 groups. Mean whole blood Co concentration was higher in the CrCo modular neck group (0.46 versus 0.26 µg/l in the Ti group, P=0.004), as was mean whole blood Ti concentration in the Ti modular neck group (1.98 versus 1.59 µg/l in the CrCo neck group, P=0.007). On the other hand, ion levels were very low, and between-group differences were of limited clinical significance (0.12 µg/l Co and 0.39 µg/l Ti), demonstrating that modular junction performance was good irrespective of the metal combination.

This study has some limitations. Whole blood metal ion measurements represent estimates of total ion load liberated from the joint and absorbed into blood, rather than the local concentrations of ions in the tissues surrounding the hip implant. Moreover, it is impossible to quantify metal ions released by passive implant surface corrosion versus modular neck junction corrosion. This is particularly important as the acetabular cup and femoral stem of all implants were composed of Ti. In contrast, Cr and Co metal ion concentrations came exclusively from modular neck stem junction corrosion or alimentary intake. Follow-up duration was relatively short. As corrosion is a gradual and continuous process, these results cannot be extrapolated over a longer period of time. The number of subjects in our study was relatively small and thus we may have missed the inclusion of problematic cases that occasionally occur. The manufacturer (MicroPort) offers maximal 40-mm femoral heads, which can be coupled with maximum 52-mm diameter Maxera cups. Men frequently require acetabular components >52 mm thus our cohort was composed almost exclusively of women. Some studies have divulged significantly higher Co metal ion concentrations in women [19].

As reported in the literature, whole blood metal ion concentrations vary widely following different orthopedic procedures. Metal ions may be released from implant surfaces by modular junction wear/corrosion or bearing wear in metal-on-metal (MoM) articulations. The present study design allowed a good quantification of Cr, Co, and Ti metal ion release from the modular neck junction. Other authors have noted metal ion release from modular junction implants, but, in all cases, this may arise from more than one component of the implant. Since metal ion liberation is caused by a series of reactions (passive, galvanic, fretting, and crevice corrosion), it is impossible to identify the exact cause.

A systematic review and case series published by Carli *et al.* [20] analyzed 24 publications, representing 776 cases of head-neck corrosion. This review determined that most of the corrosion occurred in patients undergoing THA combined with large diameter head bearings and small taper dimensions. Factors that appeared to contribute to the corrosive process included coating precipitation, mixed alloy coupling, and head-neck mismatch, indicating that a combination of mechanical processes and electrochemical dissolution phenomena are responsible for corrosion.

Vundelinckx *et al.* [21] measured metal ion levels in 19 of 306 patients who underwent THA with ABG II modular neck stems (Stryker, Kalamazoo, MI, USA) and ceramic-on-polyethylene or CoC bearing. All 19 cases were asymptomatic after at least 24 months’ follow-up. Serum Cr levels were <1.3 μg/l in the 19 cases. Serum Co level were >4 μg/l in 9/19 patients. Of the 306 patients, 1 underwent THA revision because of ARMD. This patient had 2.1 μg/l serum Cr and 7.4 μg/l serum Co.

Gofton and Beaule [22] measured Ti ion levels in the serum of 50 subjects with Profemur® TL (MicroPort) Ti modular femoral stem and Ti modular necks. Subjects were randomized to receive either a large femoral head, metal-on-metal (MoM) bearing, or 28- to 32-mm CoCr femoral head on polyethylene (MoP) within a Ti shell. Mean serum Ti levels were similar between groups at 1 year: 2.9 µg/l in MoM versus 2.8 µg/l in MoP (P=0.11). Taking into account the higher values expected in serum versus whole blood [23], these results are similar to those of the present study (1.97 µg/l). In the MoM group, Ti release may have come from corrosion of the passive femoral implant, stem-neck modular junction, or neck-head taper junction. In the MoP group, Ti release may have arisen from passive corrosion of the stem and cup or the stem-neck modular junction.

Two studies were published by Somers *et al.* [24, 25]. In the first study, 30 patients underwent THA with a hybrid THR Profemur^®^ Xm - Procotyl^®^ L prosthesis, with a Ti modular neck on a CoCr stem design and CoC bearing. After minimum follow-up of 12 months, the mean serum Co level was 1.2 µg/l in patients with short stems and 1.3 µg/l in patients with long stems, while serum Cr levels were below the detection limit of 0.5 µg/l. In the second study, the investigators evaluated 23 THA with the same THR Profemur^®^ Xm - Procotyl^®^ L prosthesis but with a CoCr modular neck. A CoCr stem and CoC bearing were implanted as in the previous study. Serum Co levels were higher (1.7 µg/l), presumably due to the CoCr modular neck. Serum Cr levels were lower (0.5 µg/l). These findings revealed that a CoCr modular neck on a CoCr stem produces significantly higher Co levels compared to a Ti modular neck on a CoCr stem. Based on these results, the authors proposed that Ti necks should not be abandoned, as CoCr necks appear to be responsible for increased Co ion release.

In the present study, whole blood Cr and Co concentrations in the Ti modular neck group were similar to those reported in people without implants [23, 26, 27] and represent normal alimentary/environmental absorption. These results were expected as implants in this group did not contain Cr or Co. Use of a CrCo modular neck elicited significantly increased Co ion levels: 0.46 µg/l versus 0.29 µg/l (p<0.01). Although, a statistically significant difference was observed, the levels encountered should not have any clinical impact. In studies reporting ARMD related to modular neck stem junction corrosion, Co concentrations were usually >7.0 µg/l [5, 6, 28]. In these cases, a Cr/Co ratio greater than 1 is frequently observed [29].

Ti ion levels measured in the present study for both CrCo or Ti necks groups are similar to those reported by many authors using non-modular Ti implants [13, 14, 23, 30-32]. Passive corrosion of Ti implants translates into whole blood levels between 1.8 μg/l and 3.1 μg/l. Such Ti levels have never been linked to Ti ARMD.

## CONCLUSION

Although, these results demonstrated that modular neck materials have an impact on metal ion release, their whole blood concentrations remained within a safe range at a mean follow-up of 20 months and were therefore not clinically meaningful. In a limited number of patients and after a short follow-up period, the current results do not show indirect signs of modular junction corrosion with either CrCo or Ti femoral necks.

## Figures and Tables

**Fig. (1):**
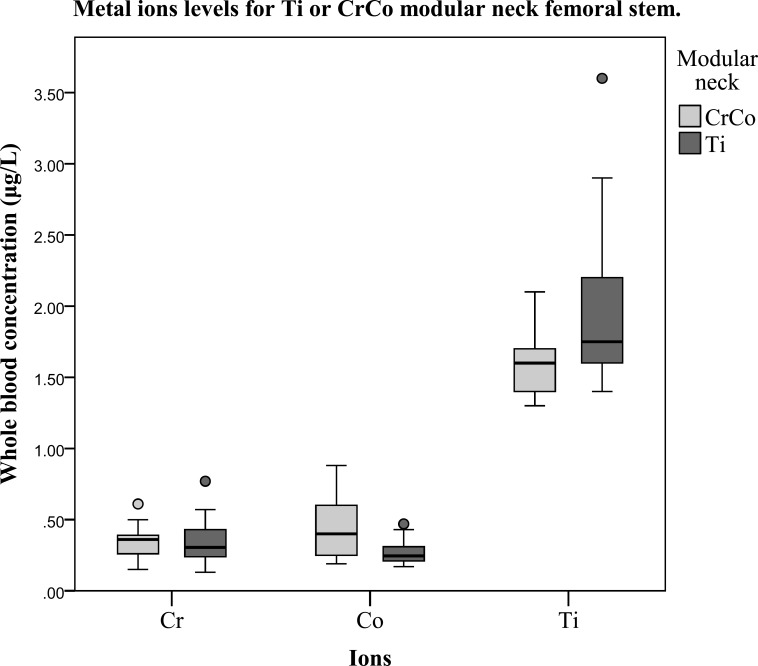
Metal ion levels at more than 1 year follow-up after large diameter head. THA as function of neck materials.

**Table 1:** Demographic data.

-	All Patients	CrCo Neck	Ti Neck	P-Values
N	36	14	22	–
Males:females	2:34	2:12	0:22	–
Mean age (min-max, SD)	53.4 (31-68, 9.9)	58.4 (33-63, 8.0)	50.2 (31-68, 9.8)	0.01
center:right side	18:18	5:9	13:9	0.17
Mean BMI (min-max, SD)	24.0 (17.6-36.1, 4.3)	23.8 (17.9-30.7, 3.8)	24.1 (17.6-36.1, 4.9)	0.89
Short:long neck length	30:6	8:6	22:0	–
Neck type Straight Var/Val 8^0 ^ ARVV1* ARVV2** 8^0 ^ DG A/R	13 14 4 4 1	8 6 0 0 0	5 8 4 4 1	–
Follow-up (min-max, SD)	20 (9-44, 7.5)	23 (9-44, 1.1)	19 (11-33, 5.9)	0.09

*Right hip: Anteverted/Varus Retroverted/Valgus

**Right hip: Anteverted/Valgus Retroverted/Varus

**Table 2:** Whole blood metal ion concentrations (µg/l) as function of modular neck materials.

	All Patients	CrCo Neck	Ti Neck	P-Values
Cr (min-max, SD)	0.34 0.13-0.77, 0.14	0.35 0.15-0.61, 0.12	0.33 0.13-0.77, 0.16	0.43
Co (min-max, SD)	0.34 0.17-0.88, 0.18	0.46 0.19-0.88, 0.23	0.26 0.17-0.47, 0.08	<0.01
Ti (min-max, SD)	1.83 1.3-3.6, 0.47	1.59 1.3-2.1, 0.22	1.98 1.4-3.6, 0.52	<0.01

**Table 3:** Clinical function scores.

Neck Type	All	CrCo Neck	Ti Neck	P-Values
UCLA pain	9.0 (2-10, 1.7)	8.8 (2-10, 2.2)	9.2 (6-10, 1.2)	0.53
UCLA walk	9.9 (8-10, 0.4)	10 (10-10, 0.0)	9.9 (8-10, 0.5)	0.42
UCLA function	9.7 (6-10, 1.0)	9.7 (6-10, 1.1)	9.7 (6-10, 1.0)	0.98
UCLA activity	7.5 (4-10, 2.0)	7.8 (4-10, 2.1)	7.3 (4-10, 1.6)	0.42
UCLA total	36.1 (26-40, 3.3)	36.3 (26-40, 3.9)	36 (30-40, 3.0)	0.80
WOMAC	5.0 (0-24, 7.1)	6.5 (0-23, 8.9)	4.1 (0-24, 5.7)	0.52
